# Managing the aftermath: complications and outcome after colorectal resection for deep infiltrating endometriosis—insights from a certified surgical endoscopy center

**DOI:** 10.1038/s41598-026-47171-9

**Published:** 2026-04-09

**Authors:** Isabelle Flammang, Ann-Kathrin Eichelmann, Sebastian Schäfer, Jan Philipp Ramspott, Alexander Bungert, Andreas Pascher, Jennifer Merten

**Affiliations:** 1 Department of General, Visceral and Transplant Surgery, University Hospital Muenster, 48149 Münster, Germany; 2 Department of Gynecology, Ludgerus-Kliniken Münster GmbH, Clemenshospital, 48153 Münster, Germany

**Keywords:** Deep infiltrating endometriosis, Bowel endometriosis, Colorectal resection, Anastomotic leakage, Endoscopic vacuum therapy, Low anterior resection syndrome, Diseases, Gastroenterology, Medical research

## Abstract

Deep infiltrating endometriosis (DIE) often affects the bowel and may necessitate colorectal resection. While protective ileostomy can reduce complications, it carries relevant morbidity. This study evaluates endoscopic complication management and long-term outcome of patients treated at a certified surgical endoscopy and endometriosis center. All patients undergoing interdisciplinary surgery for DIE (2015–2024) were retrospectively included. Preoperative sigmoidoscopy and postoperative endoscopic evaluation of anastomoses were performed routinely. Surgical approaches included shaving, excision or resection with/without ileostomy. Surgical complications, such as anastomotic leakages and stenoses, were primarily managed endoscopically. 118 women (median age: 33 years) underwent surgery: rectal shaving (25%), sigmoid (6%) and rectal resection (69%). Median anastomotic height was 10 cm, 44% received a protective ileostomy. Anastomotic leakage (8%) and stenosis (9%) were successfully managed endoscopically. Recurrence requiring reoperation occurred in 41% after shaving vs. 27% after resection. Ileostomy reversal was achieved in all cases. Postoperative continence was acceptable (LARS median: 13). General health (EQ-5D-5 L: median 70) was good at a median follow-up of 75 months. 84% would choose surgical treatment again. Colorectal resection for DIE can achieve favorable long-term outcomes in a structured interdisciplinary setting. Proactive endoscopic management supports bowel continuity despite complications. Prospective studies are needed to validate outcomes and refine patient selection.

**Supplementary Information:**

The online version contains supplementary material available at 10.1038/s41598-026-47171-9.

## Introduction

Endometriosis is a benign, chronic disease in which endometrial-like tissue grows outside the uterine cavity, leading to symptoms such as dysmenorrhea, dyspareunia, dyschezia, pelvic pain, obstipation, rectal bleeding and infertility^1^. It affects up to 10% of women of reproductive age, with a peak between 25 and 35 years and remains one of the most common gynecological disorders^2–4^.

Three subtypes are recognized: peritoneal, ovarian, and deep infiltrating endometriosis (DIE), the latter frequently affecting the rectovaginal septum, uterosacral ligaments, bladder, and bowel. Bowel involvement is found in 4–37%, most often in the sigmoid colon or rectum^5^. In up to 35% of DIE cases, gastrointestinal or urinary symptoms result from deep infiltration^6^.

Management strategies for bowel-infiltrating DIE range from conservative hormonal therapy to surgery, including rectum-conserving approaches (shaving, disc excision) and segmental colorectal resection^2^. While organ-preserving techniques are associated with lower morbidity and better functional outcomes, more radical surgery is sometimes required for symptom control^2,7–10^.

Colorectal resection in DIE carries a postoperative complication rate of approximately 20%, including rectovaginal fistula, anastomotic leakage (AL), and urinary retention^11^. Recurrence rates vary widely up to 25% depending on surgical technique^12–15^.

Around 15% of women undergoing surgery for endometriosis require a protective ileostomy^11^. In cases of DIE with bowel involvement, rectum-sparing techniques such as shaving or disc excision do not reliably prevent ileostomy^16^. The use of a temporary diverting ileostomy is supported by evidence from colorectal cancer surgery, where it significantly reduces the risk of AL and reoperation in low anterior resections^17,18^. However, in young and otherwise healthy women, the typical endometriosis population, stoma-related complications such as obstruction, retraction, abscess formation, electrolyte disturbances, and acute renal failure can significantly affect recovery. Moreover, stoma reversal is associated with a complication rate of around 20%, including AL, ileus, and wound infections^19–22^. Therefore, the decision for ileostomy is carefully considered intraoperatively, but may also become necessary postoperatively in the event of complications such as AL or rectovaginal fistula.

Despite increasing data, no consensus exists regarding the optimal surgical approach and complication management for DIE with bowel involvement. Decisions are often based on surgeon experience and institutional protocols, highlighting the need for further outcome-based research.

This study, conducted at a certified surgical endoscopy and endometriosis center at the University Hospital of Muenster, aims to evaluate outcomes of surgical treatment in patients with bowel-infiltrating DIE, with a particular focus on endoscopic complication management. By analyzing surgical data and patient-reported outcomes, we aim to identify complications and functional impairments affecting colorectal function.

## Materials and methods

### Patient cohort

All female patients who underwent interdisciplinary surgical management of endometriosis, requiring both gynecological and visceral surgical involvement, at the University Hospital of Muenster between 2015 and June 2024 were included. Patients were excluded if they did not undergo shaving or resection of the rectosigmoid area, or if endometriosis was diagnosed incidentally during surgery for unrelated conditions. Additionally, patients with a postoperative diagnosis of malignancy (e.g., ovarian or endometrial carcinoma) were excluded.

### Data collection

Written informed consent for data collection and follow-up was obtained from all patients prior to inclusion. The study was approved by institutional research committee (Ethikkommission Westfalen-Lippe, Approval No. 2019-747-f-S) and conducted in accordance with the Declaration of Helsinki. Clinical data including patient history, therapeutic background, preoperative symptoms, surgical and pathological findings, as well as follow-up data, were collected retrospectively.

### Preoperative protocol

All cases were presented in an interdisciplinary endometriosis board, including specialists in gynecology, visceral surgery, radiology, urology and pain medicine. Surgical intervention was indicated in patients with persistent symptoms and significant disease burden despite exhaustion of conservative measures, including hormonal therapy and multimodal pain management, or in cases of infertility.

Preoperative assessment included flexible sigmoidoscopy in all cases and rectal endoscopic ultrasound or pelvic MRI (Magnetic Resonance Imaging), depending on symptom severity. Urological evaluation was performed if needed.

Patients suspected of DIE with bowel involvement received detailed preoperative counseling, including discussion of possible bowel resection and protective ileostomy. Stoma site marking and education perioperatively were provided by a dedicated stoma care team. Mechanical bowel preparation was administered the day before surgery and prior surgery a single-shot antibiotic prophylaxis with cefuroxime and metronidazole was given.

### Surgical management

All surgeries were performed laparoscopically when feasible, with conversion to open surgery in cases of extensive disease or poor intraoperative conditions. Surgical approaches included shaving, disc excision or segmental bowel resection (e.g., sigmoid or low anterior rectum resection), with or without protective ileostomy. The type of intervention was tailored to intraoperative findings. The severity of DIE was assessed according to #ENZIAN and for the sake of simplicity also according to rASRM (revised American Society for Reproductive Medicine) classification^23,24^.

Anastomoses were created transanally using the double-stapling technique with a circular stapler and were tested intraoperatively via air leak test. A drain was routinely placed near the anastomosis. Stoma reversal in ileostomy patients was typically scheduled around three months postoperatively, assuming adequate anastomotic healing.

### Postoperative protocol and complication management

Postoperatively, patients underwent interdisciplinary care. They received stool softeners to support bowel function and prevent straining. Nutritional intake was gradually reintroduced based on bowel motility.

On postoperative day five, or earlier if clinical signs of e.g. AL were present, all patients underwent routine flexible sigmoidoscopy to assess anastomotic integrity. In cases where the anastomosis was confirmed to be circumferentially intact, drain removal was performed simultaneously.

Complications were systematically recorded with a focus on surgery-related events, particularly AL and anastomotic stenosis, but also included non-surgical complications such as urinary tract infections, postoperative bladder emptying disorders, pyelonephritis or pulmonary embolism.

#### Endoscopic complication management

In cases of AL, endoscopic vacuum therapy (EVT) was initiated. A customized polyurethane sponge (VivanoMed Foam, Hartmann AG) was shaped to cover the anastomotic area and connected to a VivanoTec suction device. Continuous negative pressure ranging from − 125 mmHg to −150 mmHg was applied.

In patients where AL resulted in the formation of a pelvic cavity, the sponge was placed intracavitarily and replaced every five days during follow-up sigmoidoscopies. All endoscopic procedures were typically performed without sedation. In complex cases where no protective ileostomy had been created during the initial surgery, a mechanical bowel preparation and diverting ileostomy was required prior to the initiation of endoscopic treatment. Patients with stable EVT and sufficiently short travel distance to hospital were managed on an outpatient basis.

Treatment of anastomotic stenosis was performed transanally using either balloon dilatation or manual bougienage. All endoscopic procedures were carried out by two senior endoscopic surgeons to ensure consistency of technique and assessment.

#### Surgical revision

Surgical revision was performed when EVT was considered unsuitable due to extensive ischemia, for early rectovaginal fistulas (most likely related to technical factors during low rectal anastomosis and managed surgically by secondary closure, including simultaneous protective ileostomy), or for other complications such as compartment syndrome.

### (Long-term) follow-up

All patients were instructed in pelvic floor muscle training after surgery to support anorectal function. For the long-term evaluation, the years 2015 to 2022 were considered, including a total of 98 patients who were retrospectively surveyed as part of our routine follow-up regarding quality of life and postoperative outcomes. Sixteen patients were lost to follow-up, leaving 82 patients available for analysis. Follow-up was conducted through standardized outpatient visits and structured telephone interviews to minimize data loss. Health status was assessed using the EQ-5D-5 L (European Qualitiy of Life 5 Dimensions 5 Level Version) questionnaire, and anorectal function was evaluated with the LARS (Low Anterior Resection Syndrome) score. In addition, patients were asked about the recurrence of endometriosis-related symptoms and whether they had undergone further surgery after the index procedure. Reoperations were recorded based on patient-reported information. As detailed clinical records regarding the exact indication, timing, and anatomical site of subsequent surgery were not systematically available, reported reoperations may include procedures performed for recurrent endometriosis as well as other pelvic indications, such as adhesiolysis or diagnostic interventions.

### Statistical analyses

Statistical analysis was performed using IBM SPSS Statistics 28.0, GraphPad Prism 7, and Microsoft Excel for Windows 11. Fisher’s exact test was used for categorical variables, and the Mann–Whitney U test for continuous variables. Continuous variables are presented as median with interquartile range (IQR), and categorical variables as counts and percentages. Subgroup analyses were performed based on the extent of surgical intervention and endometriosis severity according to the rASRM classification. Correlations between potential risk factors and the development of AL were evaluated using multivariate analysis. A p-value ≤ 0.05 was considered statistically significant.

### Language assistance

Language assistance for phrasing, grammar, and structure was provided using ChatGPT (OpenAI, San Francisco, CA, USA), a large language model. ChatGPT was not used to generate scientific content or interpret data. The authors critically reviewed and edited all content to ensure accuracy and appropriateness.

## Results

### Baseline patients’ characteristics and endometriosis-specific symptoms of the overall cohort

A total of 118 patients were included in the study. Their baseline characteristics, comorbidities, and symptoms are summarized in Table 1. Median age was 33 (22–54) years, and the body-mass-index (BMI) was 24,2 (18,4–45,2) kg/m^2^. 72 patients (61%) had comorbidities. The most common comorbidities were thyroid disorders (hypothyroidism or Hashimoto’s thyroiditis), gastrointestinal conditions, and central nervous system disorders (migraines and depression). 16 patients (14%) had a history of nicotine use. 55 patients (47%) were classified as ASA 1 (American Society of Anesthesiologists) and 63 patients (53%) as ASA 2 based on preoperative anesthesiologic evaluation.

The most reported symptoms were dysmenorrhea (*n* = 108, 92%) and dyspareunia (*n* = 73, 62%). In addition to menstrual cycle-independent pain, (*n* = 68, 58%), urological issues, particularly dysuria (*n* = 36, 34%), were described as well. 48 patients (41%) experienced dyschezia as the most frequent gastrointestinal symptom, with some describing cycle-dependent changes in stool quality, such as diarrhea (*n* = 19, 16%) and constipation (*n* = 38, 32%). Some patients also reported hematochezia (*n* = 29, 25%) or hematuria (*n* = 5, 4%). Every second woman suffered from an unmet desire for children, with 16 patients (14%) having a history of one or more miscarriages. 91 patients (77%) had already undergone a surgical endometriosis procedure after their initial diagnosis and were referred to our endometriosis center for further surgery due to recurrence or residual disease.

### Operative data

The surgical characteristics of the study cohort are summarized in Table 2. 114 patients (97%) were operated laparoscopically, 13 of whom (11%) required conversion to an open procedure. Only 4 patients (3%) were primarily operated openly due to multiple prior abdominal surgeries. Based on intraoperative findings using the rASRM classification, most patients had stage IV endometriosis (67%), followed by stage I (14%), stage III (13%), and stage II (6%). All patients received excision of visible endometriosis lesions, with 4 patients (3%) having minor residual endometriosis. Comprehensive adhesiolysis was necessary in 85 patients (72%), and 5 patients (4%) underwent simultaneous small bowel segment resections.

Depending on the extent of visceral surgical involvement, the patient cohort was divided into three subgroups: 30 (25%) patients underwent rectal anterior wall shaving, 7 (6%) patients underwent sigmoid resection and 81 (69%) patients underwent rectal resection. All patients were able to maintain continence, although 42 (35%) patients required temporary protective ileostomy. In cases of resecting surgery, 70 patients (59%) received end-to-end descendo-rectostomy and 18 patients (15%) received side-to-end descendo-rectostomy. In all cases, a double stapled anastomosis (size 28–32 mm) was performed. The anastomosis height was, in case of sigmoid resection, at a median of 18 cm from the anus, and in case of rectal resection, at a median of 10 cm from the anus (*p* = 0,002).

### Subgroup analysis: rectal anterior wall shaving vs. sigmoid resection vs. rectal resection

No significant differences in age, ASA classification, or BMI were found between the subgroups of shaving, sigmoid and rectal resection. There were no significant differences in comorbidities, except for relevant cardiac arrhythmias, which were more frequent in the sigmoid resection group (29% vs. 1% (rectal resection) vs. 0% (shaving); *p* = 0,009). Regarding preoperatively reported symptoms, no significant differences were found between the subgroups, except for hematochezia, which was significantly more frequent in the sigma resection subgroup (57% vs. 26% (rectal resection) vs. 13% (shaving), *p* = 0,038), and defecation pain, which was particularly reported in the rectal shaving subgroup (40% vs. 21% (rectal resection) vs. 0% (sigmoid resection), *p* = 0,034), see supplementary table S1). The rate of conversion to open surgery was significantly higher in the sigmoid resection group (43%) compared with the rectal shaving (13%) and rectal resection groups (4%, *p* = 0.023). The distribution of rASRM stages differed significantly between the three groups (*p* < 0,001), with stage IV disease being most frequent in the rectal resection group (77%), while earlier stages were more common in the rectal shaving and sigmoid resection groups (see Table 2).

### Subgroup analysis: endometriosis severity based on rASRM classification

There were also no significant differences in the severity of endometriosis based on the rASRM classification between the groups (rASRM 1–4) regarding age, ASA classification, BMI, and comorbidities. Preoperative symptom severity did not correlate with the severity of endometriosis detected intraoperatively. Of the 91 patients (77%) who had previously undergone an endometriosis-related surgery, there was a significant correlation between prior surgery and higher intraoperative endometriosis severity, as most of the previously operated patients were classified as rASRM grade 3 or 4 (*p* = 0,025, see Table 3).

### Postoperative complications

Postoperative complications are summarized in Table 2. 24% of patients experienced non-surgical complications, primarily urinary tract infections or bladder emptying disorders. In total, 9 (10%) patients had an AL, 10 (11%) had an anastomotic stenosis, and 3 (2%) developed a rectovaginal fistula. The distribution of complications was not significantly different among the subgroups, either with regard to the surgical procedure (Table 2) or disease severity (Table 3). The median postoperative hospital stay was 6 to 7 days, regardless of the surgical procedure. Postoperative intensive care unit stay was rare (see Tables 2 and 3).

### Complication management

AL occurred in 9 patients (8%). Of these, 8 patients were treated with EVT, and all leaks healed successfully after a median of 2.5 (1–9) sponge cycles. Three patients underwent creation of a secondary ileostomy to facilitate endoscopic treatment. One patient with AL due to ischemia of the descending colon required re-resection and creation of a new anastomosis, which healed without further complications. Overall, all AL resolved without permanent sequelae, with successful treatment achieved by EVT in 8 patients (although additional surgical measures (protective ileostomy) were required in selected cases) and by re-operation of the anastomosis in 1 patient. Overall, 9 patients (8%) required re-operation in the early postoperative course: besides the four aforementioned re-operations mentioned in the context of AL treatment, additional procedures included rectosigmoid resection following suture failure after rectal shaving with perforation of the anterior rectal wall (*n* = 1, uneventful postoperative course), surgical fistula closure in patients with early rectovaginal fistulas (*n* = 3, with one patient receiving a simultaneous protective ileostomy, uneventful postoperative courses), and surgical compartment release of the lower leg after rectal resection (*n* = 1).

All anastomotic stenosis were dilated once prior to ileostomy reversal. All ileostomies were successfully reversed after 3 (0,25 − 11) months, including patients who developed postoperative AL.

### Correlation of risk factors and development of AL: multivariate analysis

In multivariate analysis, no significant correlation was found between typical risk factors such as BMI, age, comorbidities, or endometriosis severity and the development of an AL. Moreover, there was no association between the placement of a protective ileostomy and the development of an AL or rectovaginal fistula. However, significantly more patients with ileostomies developed an anastomotic stenosis (21% vs. 4%, *p* = 0,038, Table 4).

### Long-term outcome

Long-term outcomes of the 82 patients who participated in the questionnaire are summarized in Table 5. Median follow-up time was 75 (38–126) months. During follow-up, 56 (68%) patients reported recurrent endometriosis-related symptoms. Among these patients, symptoms were reported as worse in 7%, unchanged in 18%, and improved in 75% compared to the preoperative situation. Patients who underwent (sigmoid or rectal) resection had a significant improvement in symptoms (*p* = 0,042). 33 (40%) patients resumed hormonal therapy. During follow-up, 24 (29%) patients reported having undergone at least one additional surgical procedure after the index operation. As detailed information on the indication for surgery was not systematically collected, these procedures may include surgery for recurrent endometriosis as well as other pelvic indications.

The EQ-5D-5 L score was 70 (20–100), with the sigmoid resection group showing a significantly better median score (85 vs. 70 (rectal resection) vs. 75 (shaving), *p* = 0,013). Every second patient who received a protective ileostomy reported stoma-related issues, particularly psychological problems. Regarding the LARS score, a total of 19 (23%) patients developed minor symptoms, and 13 (16%) patients developed major LARS, with no significant differences between the subgroups. Retrospectively, 69 (84%) patients stated that they would choose the same surgical procedure again.

## Discussion

### Baseline patients’ characteristics and endometriosis-specific symptoms of the overall cohort

The patient cohort represents a comprehensive Western population of bowel-infiltrating DIE in which the subgroups of colorectal resection (shaving, sigmoid and rectal resection) showed no significant differences regarding baseline characteristics, comorbidities or disease severity according to the rASRM classification^16^.

### Surgical approach and perioperative management

All patients were managed according to the same standardized pre- and postoperative protocol, ensuring a high degree of consistency despite the single-center study design. A considerable proportion of patients had received prior treatments, many presenting with advanced disease (rASRM stage IV), residual disease at referral, and a significant symptom burden. Nevertheless, minimally invasive surgery was feasible in most cases, and the conversion rate remained low. It must be taken into account that the advanced manifestation of DIE correlates with a higher extent of surgical trauma and might carry a higher risk for complications.

### Outcomes by surgical subgroup

The very small subgroup of patients undergoing sigmoid resection showed significantly worse outcomes, with higher rates of ileostomies and conversion. However, these patients were older, had higher BMI, and presented with more comorbidities, factors likely contributing to the surgeon’s decision to opt for an ileostomy in 50% of cases. These findings should be interpreted with caution given the very small size of this subgroup.

The overall stoma rate was with 44% high according to larger patient cohorts, including in cases with more proximal (high) anastomoses, suggesting that factors beyond anastomotic height influenced the surgical strategy intraoperatively^16^. Nevertheless, we observed that the rate of AL did not differ significantly between patients with or without a protective ileostomy. This suggests that while a diverting stoma may not prevent complications related to anastomotic healing, it can facilitate an unproblematic EVT.

The increased number of anastomotic stenosis noticed in the stoma subgroup matches current observations investigating patients after anterior resection for rectal cancer^25^. This is likely attributable to the absence of physiological defecatory stimuli during the 3 months diversion period, which may impair functional remodeling and lead to luminal narrowing.

### Complication management and reoperation rates

Despite the additional surgical trauma associated with extensive simultaneous endometriosis excision, the observed complication rate was comparable to rates reported in the current literature^8,26^, which may indicate an acceptable safety profile of this approach. In our cohort, no presacral abscesses or postoperative bleeding events were observed and the early reoperation rate was low. Reports of AL after rectal resection in patients with DIE vary between 0,6 and 21%, indicating the complexity of anastomotic healing^26–28^. In selected cases, the secondary creation of a stoma allowed preservation of the anastomosis, provided that endoscopic evaluation showed no major issues such as ischemia or complete anastomotic dehiscence. This strategy may contribute to favorable functional outcomes by avoiding unnecessary resection or revision and is supported by the 100% success rate of EVT. To our knowledge, no study to date has specifically investigated endoscopic complication management of AL in patients with endometriosis. Our data demonstrates that endoscopic treatment resulted in complete healing in all affected patients with well-perfused anastomoses. This favorable outcome is likely supported by the fact that the cohort consisted of young and otherwise healthy women.

### Functional outcomes and LARS

The reported LARS rate of 13 (0–39) points, especially in rectal resections, was relatively high compared to the literature^29^; however, these findings should be interpreted with caution, data were retrospectively obtained and based on patient-reported outcomes rather than objective functional testing such as anorectal manometry^29–31^. In addition, the LARS score was originally developed and validated for patients undergoing rectal cancer surgery and may therefore have limitations when applied to patients treated for endometriosis. Despite these limitations, most patients reported preserved continence after stoma reversal, even in cases where postoperative complications occurred. Given the potential impact of rectal surgery on bowel function, this underscores the value of a standardized complication management protocol and close interdisciplinary care. Notably, no patients were lost to early postoperative follow-up, allowing for prompt detection and effective treatment of complications.

From our perspective, delayed or non-proactive management should be viewed with caution, as late complications tend to be more challenging to treat. This is particularly relevant in young women, where the indication for surgery often includes the desire to preserve fertility; therefore, careful preoperative counseling regarding possible postoperative impairments in evacuation and continence is essential, and patients should be guided in postoperative pelvic floor training to support long-term continence.

### Hospital stay and postoperative care

The median hospital stay in our cohort was satisfactory and similar to other certified endometriosis centers, and even in cases with complications, a length of stay of approximately three weeks appears justified given the complexity of the procedures and the need for close postoperative monitoring and management^32^. We want to emphasize the possibility of outpatient therapy in case of major AL and necessity of extended EVT. Especially in young patients who recovered well from surgery with stabilized healing of the anastomosis due to EVT, outpatient therapy ensures patient self-sufficiency and reduces possible medical and psychological side effects of a prolonged hospital stay. Moreover, sponge changes were feasible without sedation in most cases, which may enhance patient comfort and acceptance of the procedure.

## Limitations

This study has several limitations. First, its retrospective and non-randomized design limits the ability to establish causal relationships and may introduce selection bias. Treatment allocation was not randomized but based on clinical and intraoperative findings, which influenced the composition and size of the study subgroups. Although baseline characteristics did not differ significantly between groups, residual confounding cannot be fully excluded. In addition, the relatively small subgroup sizes limit the statistical power of subgroup comparisons and results should therefore be interpreted with caution.

From a methodological perspective, no a priori sample size calculation was performed due to the retrospective design of the study. All eligible patients within the study period were included, resulting in a consecutive real-world cohort. As the sample size was therefore fixed, no post hoc power calculation was conducted. While the study may be underpowered to detect small differences between subgroups, it nevertheless represents one of the larger single-center experiences in this specific clinical setting.

Furthermore, the study was conducted at a single center, which may limit the generalizability of the findings to other institutions or healthcare systems. One subgroup underwent rectal shaving rather than a full rectal resection as performed in the other groups, which may affect the comparability of outcomes across cohorts. However, as the primary focus of this study was the treatment and endoscopic management of postoperative complications, differences in surgical approach are likely of limited relevance to the main conclusions.

During follow-up, 29% of patients reported having undergone at least one additional surgical procedure. This finding should be interpreted in the context of endometriosis as a chronic and often recurrent disease with a long-term course that may require repeated interventions over time. Consequently, additional surgery does not necessarily reflect failure of the initial procedure but may represent management of persistent or newly developing symptoms. As follow-up data were patient-reported, the exact indication for reoperation could not be determined and may include surgery for recurrent endometriosis, adhesions, or other pelvic symptoms.

Finally, although the follow-up period was sufficiently long and the loss to follow-up rate was relatively low (16%), outcome assessment relied on patient-reported questionnaires. While these instruments are standardized and widely validated, the use of self-administered tools may introduce a degree of subjectivity and response bias.

## Conclusions

Colorectal resection in patients with DIE can offer favorable long-term outcomes, including improved symptom control, reduced recurrence risk, preserved continence and preservation of fertility, when embedded within a perioperative, structured, interdisciplinary treatment approach. Our findings support the use of a resecting surgical strategy over local excision in selected cases, particularly when disease extent and localization warrant it. Accurate preoperative staging through multimodal diagnostics, followed by individualized surgical planning and interdisciplinary collaboration, is crucial for achieving optimal outcomes while minimizing morbidity. Furthermore, the integration of a standardized endoscopic complication management protocol allows for the effective treatment of postoperative issues and contributes to the long-term preservation of bowel continuity, even in the presence of complications such as AL or stenosis. These results underscore the importance of comprehensive, center-based individualized care models for complex endometriosis cases involving the bowel and call for further prospective studies to validate these findings and optimize patient selection. In this context, proactive complication management plays a key role in maintaining bowel function and continence, particularly in young women for whom quality of life are critical treatment goals.

**Table 1:** Patient baseline characteristics and preoperative findings of the overall cohort.

	Total		
	*n* = 118 (%)		
Age (years)	33 (22–54)	Symptoms	
		Cycle-independent pain	68 (58)
BMI (kg/m²)	24,2 (18,4–45,2)	Gynecological	
		Dysmenorrhea	108 (92)
Comorbidities	72 (61)	Dyspareunia	73 (62)
Thyroid condition	31 (26)	Hypermenorrhea	31 (26)
Nicotine abuse	16 (14)	Gastrointestinal	
Gastroenterological condition	12 (10)	Dyschezia	48 (41)
Neurological disorders	12 (10)	Constipation	38 (32)
Orthopedic condition	9 (8)	Defecation pain	29 25)
Depression/mental illness	9 (8)	Hematochezia	29 (25)
DVT/PE	7 (6)	Meteorism	23 (20)
Asthma/respiratory disease	6 (5)	Diarrhea	19 (16)
Dermatological condition	6 (5)	Emptying disorder	3 (3)
Allergy/food intolerance	6 (5)	Urological	
Other gynecological condition	5 (4)	Dysuria	36 (31)
Cardiac arrhythmia	3 (3)	Hematuria	5 (4)
Urological condition	3 (3)	Urge incontinence	2 (2)
Arterial hypertension	2 (2)		
Diabetes mellitus	1 (1)	Desire to have children	
		Yes	68 (58)
ASA-Classification			
1	55 (47)	Previous abortion	
2	63 (53)	Yes	16 (14)
Prior surgery for endometriosis			
Yes	91 (77)		

*ASA* American Society of Anaesthesiologists, *BMI* body mass index, *DVT* deep vein thrombosis, *PE* pulmonary embolism, *n* number of patients, *ns* non-significant.

**Table 2:** Surgical characteristics and postoperative course.

	Total	Rectal shaving	Sigmoid resection	Rectal resection	*p*-value
	*n* = 118 (%)	*n* = 30 (25%)	*n* = 7 (6%)	*n* = 81 (69%)	
Surgical approach					
Laparoscopy	114 (97)	30 (100)	6 (86)	78 (96)	ns
Conversion to open surgery	13 (11)	4 (13)	3 (43)	3 (4)	**0.023**
Primarily open surgery	4 (3)	0 (0)	1 (14)	3 (4)	ns
rASRM (intraoperative findings)					**<0**,**001**
1	17 (14)	11 (37)	0 (0)	6 (7)	
2	7 (6)	1 (3)	2 (29)	4 (5)	
3	15 (13)	3 (10)	3 (43)	9 (11)	
4	79 (67)	15 (50)	2 (29)	62 (77)	
Anastomotic technique					
End-to-end	70 (59)	/	7 (100)	63 (78)	ns
Side-to-end	18 (15)	/	0 (0)	18 (22)	ns
		/			
Stapler diameter (mm)					
28	6 (5)	/	0 (0)	6 (7)	ns
29	44 (37)	/	4 (57)	40 (49)	ns
31	37 (31)	/	2 (29)	35 (43)	ns
32	1 (1)	/	1 (14)	0 (0)	ns
Height of anastomosis (cm)	10 (3–19)	/	18 (10–19)	10 (3–18)	**0.002**
Postoperative complication					
Non-surgical complications	28 (24)	4 (13)	1 (14)	23 (28)	ns
Anastomotic leakage	9 (8)	/	1 (14)	8 (10)	ns
Anastomotic stenosis	10 (9)	/	1 (14)	9 (11)	ns
Rectovaginal fistula	3 (3)	1 (3)	0 (0)	2 (2)	ns
Complication management					
EVT	8 (7)	0 (0)	1 (14)	7 (9)	ns
Changes (5 days/cycle)	2,5 (1–9)	0 (0)	3 (1–3)	2 (1–9)	ns
Dilatation	8 (7)	0 (0)	1 (14)	7 (9)	ns
Re-operation	9 (8)	2 (7)	1 (14)	7 (9)	ns
Protective ileostomy					**0.001**
Yes	42 (36)	3 (10)	3 (43)	36 (44)	
Stoma reversal (months)	3 (0,25-11)	5 (3–7)	3 (2–5)	3,5 (0,25-11)	ns
Postoperative hospital stay (days)	7 (2–26)	6 (2–11)	6 (5–17)	7 (3–26)	ns
Intensive care unit	0 (0–4)	0 (0–1)	0 (0)	0 (0–4)	ns

*n* number of patients, *ns* non-significant, *EVT* endoscopic vacuum therapy, *rASRM* revised American Society of Reproductive Medicine.

**Table 3:** Patient baseline characteristics and postoperative course related to rASRM classification.

	Total	rASRM 1	rASRM 2	rASRM 3	rASRM 4	*p*-value
	*n* = 118 (%)	*n* = 17 (14%)	*n* = 7 (6%)	*n* = 15 (13%)	*n* = 79 (67%)	
Age (years)	33 (22–54)	34 (28–41)	33 (27–41)	29 (22–43)	33 (23–54)	ns
BMI (kg/m²)	24,2 (18,4–45,2)	21,6 (18,6–36,1)	33,9 (19,1–45,2)	22,9 (19,4–39,9)	24,4 (18,4–41,8)	ns
Comorbidities						ns
Yes	72 (61)	8 (47)	4 (57)	13 (87)	47 (59)	
ASA-Classification						ns
1	55 (47)	8 (47)	3 (43)	7 (47)	37 (47)	
2	63 (53)	9 (53)	4 (57)	8 (53)	42 (53)	
Disease severity						ns
0–4 symptoms	66 (56)	11 (65)	5 (71)	6 (40)	44 (56)	
5–10 symptoms	52 (44)	6 (35)	2 (29)	9 (60)	35 (44)	
Desire to have children						ns
Yes	68 (58)	10 (59)	3 (43)	9 (60)	46 (58)	
Previous abortion						ns
Yes	16 (14)	3 (18)	1 (14)	2 (13)	10 (13)	
Previous operation due to endometriosis						**0.025**
Yes	91 (77)	9 (53)	4 (57)	12 (80)	66 (84)	
Postoperative complication						
Non-surgical complications	28 (24)	3 (18)	1 (14)	4 (27)	20 (25)	ns
Anastomotic leakage	9 (8)	1 (6)	0 (0)	2 (13)	6 (8)	ns
Anastomotic stenosis	10 (9)	0 (0)	1 (14)	3 (20)	6 (8)	ns
Rectovaginal fistula	3 (3)	1 (6)	0 (0)	0 (0)	2 (3)	ns
Protective ileostomy						ns
Yes	42 (36)	5 (29)	5 (71)	5 (33)	27 (34)	
Stoma reversal (months)	3 (0,25-11)	4 (2–8)	3 (2–4)	4 (2–7)	3,5 (0,25-11)	ns
Postoperative hospital stay (days)	7 (2–26)	6 (2–25)	7(4–13)	8 (2–21)	7 (3–26)	ns
Intensive care unit	0 (0–4)	0 (0–4)	0 (0)	0 (0)	0 (0–1)	ns

*n* number of patients, *ns* non-significant, *BMI* body mass index, *ASA* American Society of Anaesthesiologists, *rASRM* revised American Society of Reproductive Medicine.

**Table 4:** Comparison of postoperative complications with or without protective ileostomy after rectosigmoid resection.

	Total *n* = 88 (%)	Protective ileostomy *n* = 39 (44%)	No protective ileostomy *n* = 49 (56%)	p-value
Postoperative complication				
Anastomotic leakage	9 (10)	5 (13)	4 (8)	ns
Anastomotic stenosis	10 (11)	8 (21)	2 (4)	**0.038**
Rectovaginal fistula	2 (2)	1 (3)	1 (2)	ns

*n* number of patients, *ns* non-significant.

**Table 5:** Follow-up and long-term outcome.

	Total	Rectal shaving	Sigmoid resection	Rectal resection	*p*-value
	*n* = 82* (%)	*n* = 22 (27%)	*n* = 5 (6%)	*n* = 55 (67%)	
Follow-up (months)	75 (38–126)	103 (76–126)	54 (45–73)	62 (38–124)	**<0**,**001**
Protective ileostomy					**0.021**
Yes	30 (37)	3 (13)	2 (40)	25 (45)	
Problems with ileostomy					ns
Yes	15 (50)	1 (33)	3 (100)	11 (46)	
Endometriosis related symptoms					ns
Yes	56 (68)	18 (82)	3 (60)	35 (64)	
Symptoms					**0.042**
Worse	6 (7)	3 (14)	0 (0)	3 (5)	
Equal	15 (18)	8 (36)	0 (0)	7 (13)	
Better	61 (75)	11 (50)	5 (100)	45 (82)	
Necessitiy of hormonal therapy					ns
Yes	33 (40)	7 (32)	2 (40)	24 (44)	
Re-operation					ns
Yes	24 (29)	9 (41)	0 (0)	15 (27)	
LARS					ns
No	50 (61)	15 (68)	3 (60)	32 (58)	
Minor	19 (23)	6 (27)	2 (40)	11 (20)	
Major	13 (16)	1 (5)	0 (0)	12 (22)	
Would repeat the surgical procedere					ns
Yes	69 (84)	19 (87)	4 (80)	46 (84)	
EQ-5D-5 L (points)	70 (20–100)	75 (20–100)	85 (65–90)	70 (25–95)	**0.013**
Unmet desire to have children					ns
Yes	44 (54)	10 (45)	3 (60)	31 (56)	

*n* number of patients, *ns* non-significant, *LARS* Low Anterior Resection Syndrome, *EQ-5D-5 L* European Qualitiy of Life 5 Dimensions 5 Level Version.

## Supplementary Information

Below is the link to the electronic supplementary material.

Supplementary Material 1

## Data Availability

All data generated and/or analyzed during this study are included in this published article.
